# Proteinuria is accompanied by intratubular complement activation and apical membrane deposition of C3dg and C5b-9 in kidney transplant recipients

**DOI:** 10.1152/ajprenal.00300.2021

**Published:** 2021-12-20

**Authors:** Gustaf L. Isaksson, Marie B. Nielsen, Gitte R. Hinrichs, Nicoline V. Krogstrup, Rikke Zachar, Heidi Stubmark, Per Svenningsen, Kirsten Madsen, Claus Bistrup, Bente Jespersen, The CONTEXT Study Group, Henrik Birn, Yaseelan Palarasah, Boye L. Jensen

**Affiliations:** ^1^Department of Molecular Medicine-Cardiovascular and Renal Research, University of Southern Denmark, Odense, Denmark; ^2^Department of Nephrology, Odense University Hospital, Odense, Denmark; ^3^Department of Renal Medicine, Aarhus University Hospital, Aarhus, Denmark; ^4^Department of Biomedicine, Aarhus University, Aarhus, Denmark; ^5^Department of Nephrology, Rigshospitalet, Copenhagen, Denmark; ^6^Department of Pathology, Odense University Hospital, Odense, Denmark; ^7^Department of Clinical Research, University of Southern Denmark, Odense, Denmark; ^8^Department of Clinical Medicine, Aarhus University, Aarhus, Denmark; ^9^Department of Molecular Medicine-Cancer and Inflammation Research, University of Southern Denmark, Odense, Denmark

**Keywords:** complement system, kidney, kidney transplant recipient, proteinuria, urine extracellular vesicle (exosome)

## Abstract

Proteinuria predicts accelerated decline in kidney function in kidney transplant recipients (KTRs). We hypothesized that aberrant filtration of complement factors causes intraluminal activation, apical membrane attack on tubular cells, and progressive injury. Biobanked samples from two previous studies in albuminuric KTRs were used. The complement-activation split products C3c, C3dg, and soluble C5b-9-associated C9 neoantigen were analyzed by ELISA in urine and plasma using neoepitope-specific antibodies. Urinary extracellular vesicles (uEVs) were enriched by lectin and immunoaffinity isolation and analyzed by immunoblot analysis. Urine complement excretion increased significantly in KTRs with an albumin-to-creatinine ratio of ≥300 mg/g compared with <30 mg/g. Urine C3dg and C9 neoantigen excretion correlated significantly to changes in albumin excretion from 3 to 12 mo after transplantation. Fractional excretion of C9 neoantigen was significantly higher than for albumin, indicating postfiltration generation. C9 neoantigen was detected in uEVs in six of the nine albuminuric KTRs but was absent in non-albuminuric controls (*n* = 8). In C9 neoantigen-positive KTRs, lectin affinity enrichment of uEVs from the proximal tubules yielded signal for iC3b, C3dg, C9 neoantigen, and Na^+^-glucose transporter 2 but only weakly for aquaporin 2. Coisolation of podocyte markers and Tamm–Horsfall protein was minimal. Our findings show that albuminuria is associated with aberrant filtration and intratubular activation of complement with deposition of C3 activation split products and C5b-9-associated C9 neoantigen on uEVs from the proximal tubular apical membrane. Intratubular complement activation may contribute to progressive kidney injury in proteinuric kidney grafts.

**NEW & NOTEWORTHY** The present study proposes a mechanistic coupling between proteinuria and aberrant filtration of complement precursors, intratubular complement activation, and apical membrane attack in kidney transplant recipients. C3dg and C5b-9-associated C9 neoantigen associate with proximal tubular apical membranes as demonstrated in urine extracellular vesicles. The discovery suggests intratubular complement as a mediator between proteinuria and progressive kidney damage. Inhibitors of soluble and/or luminal complement activation with access to the tubular lumen may be beneficial.

## Graphical abstract

**Figure:**
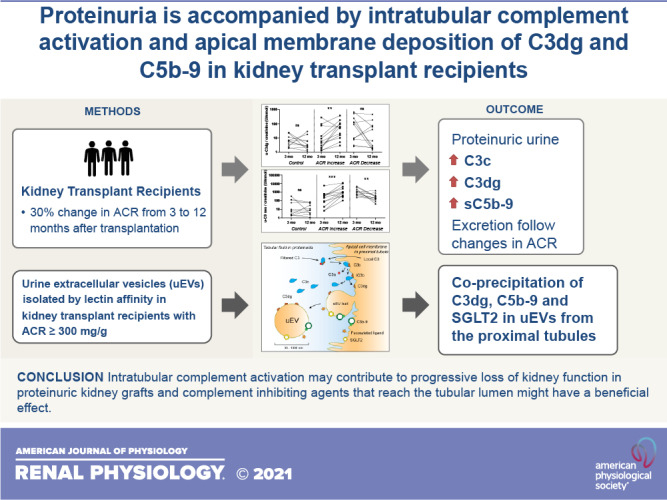

## INTRODUCTION

Proteinuria is a hallmark of glomerular filtration barrier injury in both native and transplanted kidneys and is an independent prognostic factor for decline in kidney function, cardiovascular disease, and death (1–3). Urine excretion of complement factors (complementuria) was first documented in relation to proteinuria in animal models of kidney transplantation more than 50 yr ago (4). Proteinuria is associated with a higher risk of kidney failure, and changes in albuminuria predict kidney outcome in chronic kidney disease (CKD) (5–8). Experimental evidence suggests that one or several filtered proteins are directly causing progressive kidney injury (9–11).

The complement system is activated by immunoglobulins (classical pathway) by recognition of carbohydrate patterns (lectin pathway) and by continuous but tightly regulated hydrolyzation and tick over of C3 (alternative pathway) (12, 13). The rate-limiting step in complement activation is C3 conversion, which can be quantified by measuring the C3 split products C3c and C3dg (14, 15). Downstream activation and membrane attack involves C5b-8 formation on a cell surface and the incorporation of 10–18 C9 molecules in a ring structure, forming a cytolytic pore in the cell membrane (16, 17). Urine extracellular vesicles (uEVs) are released from all segments of the nephron to the tubular fluid (18–20) and are attractive for studying tubular apical membranes. Complement attack has been studied in platelet and endothelial extracellular vesicles (21), and C3/iC3b and C9 have been detected in uEVs from patients with autosomal dominant polycystic kidney disease (22).

Proteolytically active complement factors B, D, and I (needed for C3 activation and C3dg deposition) are present in urine from patients with nephrotic syndrome but not in urine from healthy individuals (23). Increased urinary C3dg excretion has been observed in proteinuria independent of underlying glomerular disease (24). Rats with C6 depletion (and unable to form C5b-9) were protected from tubulointerstitial injury after puromycin aminonucleoside-induced nephrotic proteinuria (25) but not from nonproteinuric kidney injury (26). This observation links proteinuria and aberrant filtration of complement to kidney injury, as kidney-derived local C6 alone is not sufficient for C5b-9 formation and deposition in rats (27). Proximal tubular cells exposed to 100 to 440-kDa plasma proteins (expected size of native complement) but not 30- to 100-kDa plasma proteins had an increased apoptotic response (28), activated serum complement, and induced C5b-9 deposition in vitro (29). One study showed that high-molecular-weight proteinuria is more strongly associated with decline in kidney function than proteinuria dominated by proteins of <100 kDa (30).

Together, these observations led us to test the following hypotheses using urine and plasma samples from kidney transplant recipients (KTRs) and nontransplanted patients: *1*) complement factors are filtered from plasma and activated in the tubular lumen in proteinuria, *2*) complement activation split products are detectable in proteinuric urine, and *3*) complement deposition can be identified on uEVs representing tubular apical membranes.

## MATERIALS AND METHODS

### Study Populations

The study included samples from three different patient cohorts.

#### Cohort 1.

Biobanked EDTA-plasma and spot urine samples from KTRs were collected as part of a published, cross-sectional observational study (31). KTRs aged 18−75 yr old and graft age >12 mo were included between February and November 2016 from the outpatient clinic at the Department of Nephrology, Odense University Hospital (Odense, Denmark). Patients treated with mineralocorticoid antagonists were excluded. KTRs were included in the present study based on a urine albumin-to-creatinine ratio (ACR) of <30 mg/g (*n* = 18) or ≥300 mg/g (*n* = 19). Urine samples were divided in two fractions at collection. The first was intended for uEV analysis and treated with one cOmplete tab (Roche, Basel, Switzerland) per 50 mL, and the other was aliquoted without protease inhibition after centrifugation at 13,000 *g* for 1 min. All samples were then frozen and stored at −80°C. The study was approved by the Ethics Committee of the Region of Southern Denmark (Project ID: S-20150015 with amendment December 3, 2018) and the Danish Data Protection Agency (ID: 2008-58-0035).

#### Cohort 2.

Biobanked EDTA-plasma and spot urine samples (not protease inhibited) collected from KTRs as part of a published, prospective, multicenter, randomized controlled trial (the CONTEXT study) of 225 patients undergoing deceased donor kidney transplantation (www.ClinicalTrials.gov identifier: NCT01395719) (32). The study evaluated the effect of remote ischemic preconditioning in renal transplantation and included patients of ≥18 yr of age receiving a renal transplantation at four centers (Aarhus University Hospital, Aarhus, Denmark; Sahlgrenska University Hospital, Gothenburg, Sweden; University Medical Center Groningen, Groningen, The Netherlands; and Erasmus MC, University Medical Center, Rotterdam, The Netherlands). Time points were chosen to allow KTRs to recover from transplantation, minimize local and systemic inflammation related to transplantation, and for kidney function to get closer to a steady state, but still allow for sufficient follow-up time. Blood pressure measurements in the CONTEXT study are office recordings, but home measurements were used occasionally. All KTRs were on triple immunosuppressive therapy with tacrolimus, mycophenolate mofetil, and prednisolone at discharge from the hospital. The following three groups of KTRs were selected from the CONTEXT study database based on albuminuria at 3 and 12 mo posttransplantation: *1*) patients with an ACR of <30 mg/g at both time points (*n* = 11), *2*) patients with a ≥30% increase in ACR from 3 to 12 mo to at least 250 mg/g (*n* = 12), and *3*) patients with an ACR of at least 250 mg/g at 3 mo and a ≥30% decrease at 12 mo (*n* = 11; Supplemental Fig. S1; all Supplemental Material is available at https://doi.org/10.6084/m9.figshare.15097095.v1). Subgroup characteristics are shown in Table 1. The study was approved by the national agencies in Denmark, Sweden, and The Netherlands and by the local ethics committees (Denmark: 31894 November 2011, The Netherlands: 2013/141) and the regional ethics board (Sweden) as well as the Danish Data Protection Agency (Denmark: J. No. 2011-41-6477).

**Table 1.** Patient characteristics (CONTEXT study subgroups)

	ACR < 30 mg/g (*n* = 11)	ACR Increase ≥ 30% (*n* = 12)	ACR Decrease ≥ 30% (*n* = 11)
Recipient age, yr	53.1 (38.1–60.6)	64.4 (57.3–71.4)	56.0 (44.8–63.6)
Recipient sex (male/female), *n* (%)	4/7 (36/64)	10/3 (83/17)	9/2 (81/19)
Weight, kg	79 (65–81)	76 (71–85)	76 (74–87)
Body mass index, kg/m^2^	25 (24–29)	26 (24–29)	26 (25–27)
ACR, mg/g			
3 mo	19.0 (12.1–29.4)	90.8 (52.7–144)	333 (265–889)
12 mo	17.6 (13.7–21.2)	516 (275–889)‡	102 (68.5–168)‡
Estimated glomerular filtration rate, mL/min/1.73 m^2^			
3 mo	52 (44–67)	29 (23–46)*	44 (35–46)
12 mo	56 (41–67)	33 (26–55)†	41 (33–46)
Blood pressure, mmHg			
3 mo			
Systolic	143 (132–151)	149 (130–158)	145 (138–157)
Diastolic	88 (76–93)	80 (76–82)	87 (70–100)
12 mo			
Systolic	132 (117–142)	154 (150–161)*	131 (124–136)†
Diastolic	80 (66–85)	83 (80–93)	84 (79–85)
Antihypertensive medication (3 mo), *n* (%)			
ACE inhibitors	3 (27)	1 (11)	5 (56)
Calcium antagonists	8 (73)	6 (67)	7 (78)
Diuretics	6 (55)	1 (11)	5 (56)
β-Blockers	6 (55)	2 (22)	7 (78)
Others	0 (0)	2 (22)	1 (11)
No treatment	1/11 (9)	1/9 (11)	1/9 (11)
Antihypertensive medication (12 mo), *n* (%)			
ACE inhibitors	2 (18)	4 (33)	3 (38)
Calcium antagonists	9 (81)	6 (50)	6 (75)
Diuretics	4 (36)	2 (17)	4 (50)
β-Blockers	7 (64)	5 (42)	6 (75)
Others	0 (0)	1 (8)	0 (0)
No treatment	1/11 (9)	1/12 (8)	0/8 (0)
Rejection status, *n* (%)			
Rejections before discharge	0 (0)	1 (8)	1 (9)
Rejections at 1−3 mo	0 (0)	5 (42)	0 (0)
Rejections at 3−12 mo	0 (0)	0 (0)	0 (0)
Total	0/11 (0)	6/12 (50)	1/11 (9)

Shown are characteristics of kidney transplant recipient subgroups selected from the CONTEXT study database. Data are presented as medians with interquartile ranges or number of subjects (*n*) with percentages. Blood pressure measurements and medication registration are missing for ≤3 kidney transplant recipients. Compared with control at the same time point (unpaired): *P < 0.01; 12 mo compared with 3 mo (paired): †*P* < 0.05 and ‡*P* < 0.001. ACE, angiotensin-converting enzyme; ACR, albumin-to-creatinine ratio.

#### Cohort 3.

Urine from nontransplanted patients (*n* = 10) with an ACR of ≥300 mg/g and control urine with an ACR of <30 mg/g (*n* = 6) were collected in a cross-sectional study of damage mechanisms in proteinuria. Patients aged 18−75 yr old were included at the outpatient clinic at the Department of Nephrology, Odense University Hospital, and control urine was collected from laboratory staff volunteers. Patients treated with amiloride, aldosterone, or aldosterone receptor blockers were excluded. Baseline clinical data and medical history, including kidney disease diagnosis, were not recorded. Patient urine samples were added one cOmplete tab (Roche) per 50 mL directly after voiding and stored at −80°C. The study was approved by the Ethics Committee of the Region of Southern Denmark (Project-ID: S-20160020) and the Danish Data Protection Agency (ID: 2018-10-058).

All participants gave written informed consent, and the studies were performed in accordance with the Declaration of Helsinki.

### Enzyme-Linked Immunosorbent Assays

Established in-house sandwich ELISAs were used to measure C3c (14), C3dg (15, 33), soluble (s)C5b-9-associated C9 neoantigen, and mannose-binding lectin (MBL) in plasma and urine samples (34, 35). Plates were coated with primary antibody diluted in 100 µL carbonate coating buffer (15 mM Na_2_CO_3_ and 35 mM NaHCO_3_, pH 9.6). C3c was measured using previously validated mouse monoclonal α-C3c F1-4 (2.0 µg/mL) and biotinylated rabbit polyclonal α-C3c (1:3,000, Cat. No. A0062, Agilent-DAKO, Santa Clara, CA) (14), with minor modifications to development. C3dg was measured using rat monoclonal α-C3dg 15-39-06 (2.5 µg/mL) (15), previously validated to be specific for the C3dg fragment (36), and detected with biotinylated rabbit polyclonal α-C3d (1:500, Cat. No. A0063, Agilent-DAKO), as previously described (33) with minor modifications to development. sC5b-9-associated neoantigen was measured using mouse monoclonal α-C9 neoantigen WU13-15 [2.0 µg/mL, Cat. No. HM2264 (34), Hycult Biotech, Uden, The Netherlands] and pooled biotinylated mouse monoclonal α-C9 antibodies (1:500 each of 8-12-67 and 8-12-71, Bioporto, Hellerup, Denmark) in a Tris-buffered saline (TBS) system. MBL was measured using mouse monoclonal α-MBL HYB-131 (2.0 µg/mL, Statens Serum Institut, Copenhagen, Denmark) and biotinylated mouse monoclonal α-MBL HYB-131 (1:500) (35). Urine samples were concentrated four- to ninefold with 30-kDa cutoff, Amicon Ultra-0.5 Centrifugal filter devices (Merck-Millipore, Darmstadt, Germany) before MBL analysis. Samples were thawed on ice or at 4°C and centrifuged at 1,000 *g* (plasma) or 10,000 *g* (urine) for 5 min before analysis (5415 R, Eppendorf, Hamburg, Germany). Hu-serum activated by incubation at 37°C for 7 days (C3c and C3dg and sC5b-9) and a citrate plasma pool (MBL) were used as standard in twofold dilution. Standards were defined as 1,000 U/mL for all assays. Biotin conjugation was performed as previously described (37).

All plates were developed with streptavidin-conjugated horseradish peroxidase (Strep-HRP; Invitrogen, Carlsbad, CA) diluted 1:3,000 and 3,3′,5,5′-tetramentylbenzidine (1-Step Ultra TMB, Pierce, Thermo Fischer Scientific, Rockford, IL) and stopped with 0.2 M H_2_SO_4_. Plates were read at 450 nm on a Vmax microplate reader (Molecular Devices, San Jose, CA). Intra-assay variation [average coefficient of variance (cv)% of 30–40 measurements within the standard curve] was <10%, and interassay variation (cv% of the average mean plasma pool sample on 4−6 plates) was <15% for all assays. Measurements below the detection range were defined as 1/2 of the lowest value on the standard curve. The effects of freeze-thaw cycles on C3dg and C5b-9 assays were tested in urine samples, protease inhibited with one cOmplete tab (Roche) per 50 mL, with no changes observed after up to 11 cycles (Supplemental Fig. S2).

The fractional excretion (FE) of protein was normalized to the FE of albumin {FE_(_*_x_*_/albumin)_ = (u[*x*] × p[albumin])/(p[*x*] × u[albumin])}, providing a rough estimate of aberrant filtration versus tubular handling.

### Isolation of uEVs

uEVs were isolated either by ultracentrifugation at 220,000 *g* for 100 min as previously described (31) or by polyethylene glycol (PEG) precipitation (38). To increase uEV purity, resuspended PEG-precipitated uEVs were subjected to lectin affinity isolation for nephron segment-specific isolation of uEVs (18) and immunoaffinity isolation for C3dg and native C9 and secondary centrifugation. PEG also precipitates larger proteins, but secondary centrifugation has been shown to increase extracellular vesicle purity from soluble proteins substantially (38) and centrifugation at 10,000 *g* to isolate microvesicles >100 nm (39). Ultracentrifuged samples were normalized to urine creatinine by relative dilution. PEG, lectin affinity, and immunoaffinity uEV isolates were normalized to equal volume loaded in each well.

#### PEG precipitation.

Five milliliters of urine from six KTRs with albuminuria and positive for C9 neoantigen were thawed at 4°C and centrifuged for 15 min at 5,000 *g* (5910 R, Eppendorf) to remove cells and cellular debris. For each sample, the supernatant was moved to a new tube with the addition of 5 mL of freshly made 16% PEG6000 (Sigma-Aldrich, St. Louis, MO) with 1 M NaCl. Samples were incubated overnight at 4°C and centrifuged again for 15 min at 5,000 *g* in a precooled centrifuge (5910 R, Eppendorf) at 4°C. The supernatant was removed, and tubes were allowed to dry upside down for 10 min. The pellet was then resuspended in 500 µL PBS and mixed thoroughly.

#### Lectin affinity enrichment.

Lectin affinity enrichment was performed in PEG precipitated samples to enrich for uEVs from proximal tubules and collecting duct principal cells. Biotinylated *Lotus tetragonolobus* lectin (LTL; 100 µg, Vector Laboratories, Burlingame, CA), biotinylated *Dolichos biflorus* agglutinin (DBA; 100 µg, Vector Laboratories), or an equal volume PBS (50 µL) were added to 200 µL of PEG precipitate in 1.5-mL tubes and incubated in a rotator at 4°C for 60 min. Streptavidin magnetic beads (50 µL, Pierce, Thermo Fisher Scientific) were added to each tube and incubated under rotation at 4°C for another 60 min. The magnetic beads were removed to the side of the tubes by magnetic force, and the unbound uEV fraction was removed and saved. To wash the beads, the magnet was removed; ∼1.2 mL PBS was added to the tubes, and the samples were mixed. The beads were then removed to the side by a magnet again and the fluid was discarded. The washing procedure was repeated three times. LTL uEVs were eluted with 100 µL of 0.1 M fucose (Vector Laboratories), and DBA uEVs were eluted with 0.2 M *N*-acetylgalactosamine (Vector Laboratories) for 30 min. Beads were again moved to the side by a magnet and the eluates were moved to new tubes.

#### Immunoaffinity isolation.

Immunoaffinity isolation was performed by incubating 100 µL PEG precipitate (pool from 6 C9 neoantigen-positive KTRs) with either biotinylated α-C3dg 15-39-06 (5 µg/mL) or pooled biotinylated α-native C9 (4 µg/mL each of 8-12-67 and 8-12-71) in PBS (total volume: 500 µL) in a rotator at 4°C for 60 min. Samples were then incubated with streptavidin magnetic beads (Pierce, Thermo Fisher Scientific) for another 60 min. The unbound fraction was removed and saved, and the beads washed three times as described above, eluted for 30 min in 0.5% citric acid, and normalized with Tris (pH 7.4).

#### Secondary centrifugation.

One hundred microliters of the PEG-precipitate uEV pool was diluted in 1.2 mL PBS and centrifuged at 16,000 *g* in a tabletop centrifuge (5415 R, Eppendorf) for 60 min. The supernatant was removed, and the pellet was resuspended in 100 µL PBS and mixed thoroughly. Tertiary centrifugation was performed by adding 25 µL of the resuspended pellet to two tubes with 1.2 mL PBS and centrifuging at either 5,000 *g* (negative control) or 10,000 *g* for 30 min. The supernatant was again removed from the tubes, and the pellets were resuspended in 50 µL PBS.

Samples were either analyzed immediately or stored at −20°C for a shorter period of time if analysis could not be undertaken right away.

### Immunoblot Analysis

uEVs were analyzed by Western blot analysis using monoclonal antibodies against complement split products and uEV markers. Electrophoresis was performed on Bolt 4–12% bis-Tris Plus gels and MES running buffer using the NuPAGE System (Invitrogen). Samples were mixed with lithium dodecyl sulfate (LDS) NuPage sample buffer (4×) or LDS with 10% β-mercaptoethanol (Sigma-Aldrich) or 0.1 M DTT (Sigma-Aldrich) and heated to 85°C for 10 min. A Novex Sharp prestained ladder (Invitrogen) was included as a molecular weight marker. Gels were blotted onto PVDF membranes (P 0.45 Amersham, Hybond, GE Healthcare Life Sciences, Chicago, IL) or 0.2-µm Trans-Blot Turbo transfer membranes (Bio-Rad Laboratories, Hercules, CA) and blocked with PBS with 0.05% Tween 20 (PBStw) with 3% no-fat skim milk (Sigma-Aldrich). Membranes were incubated with the following mouse monoclonal antibodies: α-HuC9 WU13-15 (0.25 µg/mL, Hycult) (34), a-HuCD9 (1:1,000, clone 209306, R&D Systems, Minneapolis, MN), α-CD63 MX-49.129.5 (1:500, Cat. No. sc-5275, Santa Cruz Biotechnology, Dallas, TX), α-aquaporin 2 (AQP2; 1:500, Cat. No. sc-515770, Santa Cruz Biotechnology), α-Na^+^-glucose transporter 2 (α-SGLT2; 1:500, D-6, Cat. No. sc393350, Santa Cruz Biotechnology), α-podocalyxin-like antibody (1:500, 3D3, Novus Biological, Littleton, CO), α-complement receptor 1 (CR1/CD35; 1:1,000, 9H3, BioLegend, San Diego, CA), α-HuC3c (1 µg/mL, F1-4) (14), α-chicken C3 (IgG control, 1 µg/mL, Hyb 12-01) (40), rabbit α-tumor susceptibility gene 101 protein (TSG101; 1:1,000, Cat. No. ab30871, Abcam, Cambridge, UK), sheep α-HuTamm-Horsfall glycoprotein (THP/uromodulin; 1:4,000, Biotrend Chemikalien, Cologne, Germany), and biotinylated monoclonal rat α-HuC3dg IgM 13-39-06 (1 µg/mL) (15). C3-depleted serum A314 (Complement Technology, Tyler, TX) was used to validate biotinylated α-C3dg. Membranes were subsequently incubated with horseradish peroxidase-conjugated goat anti-mouse antibody (Cat. No. P0447, 1:2,000, Agilent-DAKO), goat anti-rabbit antibody (Cat. No. P0448, 1:2,000, Agilent-DAKO), rabbit anti-sheep antibody (Cat. No. P0163, 1:2,000, DAKO, Glostrup, Denmark), or Strep-HRP (1:3,000, Invitrogen), developed with enhanced chemiluminescence (Perkin-Elmer, Waltham, MA), and recorded with a ChemiDocXRS+ using Imagelab software v.6 (Bio-Rad Laboratories). EDTA-plasma or homogenized human kidney tissue (Regional Ethics Committee Approval S-20140159) were used as positive controls.

### Immunohistochemistry

Tissue was obtained from patients undergoing nephrectomy due to renal cancer at the Department of Urology, Odense University Hospital. All patients gave informed oral and written consent; the use was approved by the Institutional Review Board/Regional Ethics Committee (S-20140159) and the National Data Protection Authority (2012-58-0018). Immunohistochemistry was performed on paraffin-embedded nontumorous sections from cancer nephrectomies with or without proteinuria verified by dipstick, as previously described with minor modifications (41). Sections were blocked with TBS with Tween 20 with 5% skim milk and stained for C3dg with polyclonal rabbit α-HuC3d (Cat. No. A0063, DAKO-Agilent) diluted 1:100 or for C9 neoantigen with α-HuC9 WU13-15 (Hycult) diluted 1:1,000 overnight. Rabbit IgG (Cat. No. X0903, DAKO-Agilent) diluted 1:645 or mouse IgG (Cat. No. X0931, DAKO-Agilent) diluted 1:1,220 were used for negative IgG controls. Secondary antibodies were goat anti-rabbit IgG horseradish peroxidase (Cat. No. P0448, DAKO-Agilent) or goat anti-mouse IgG horseradish peroxidase (Cat. No. P0047, DAKO-Agilent), both diluted 1:2,000. Staining was visualized with 3,39-diaminobenzidine + substrate (Cat. No. K3468, Dako) and counterstained with hematoxylin (Sigma-Aldrich). An Olympus BX51 microscope (Olympus, Tokyo, Japan) with a DP26 camera using cellSens software (Olympus) was used to take pictures.

#### Standard laboratory analyses.

Standard clinical laboratory analyses were performed at the Department of Clinical Biochemistry and Pharmacology, Odense University Hospital, or the Department of Clinical Biochemistry, Aarhus University Hospital. Estimated glomerular filtration rate (eGFR) was calculated from plasma creatinine by the CKD Epidemiology Collaboration (CKD-EPI) equation without correction for race.

### Statistical Analyses

Group differences were analyzed by an unpaired Student’s *t* test. A paired *t* test was used to compare changes over time in the CONTEXT study cohort. Tests were performed on log-transformed data if data were log-normally distributed. A one-sample *t* test was used to compare the FE difference against 1. Correlations were assessed by Spearman’s correlations. Mann-Whitney or Wilcoxon-matched pairs signed-rank tests were used if normal distribution could not be achieved by log transformation. One-way ANOVA or a mixed model was used to compare multiple groups. Prism 8 (GraphPad Software, San Diego, CA) was used for statistical analyses. *P* < 0.05 was considered statistically significant.

### EV-TRACK

We have submitted all relevant data of our experiments to the EV-TRACK knowledgebase (EV-TRACK ID: EV210305) (42).

## RESULTS

### Patient Characteristics

In the cross-sectional cohort, KTRs with albuminuria had higher blood pressure and received more antihypertensive medication, as previously published (31). The median urine ACR with interquartile range (IQR) was 1,180 (1,032–2,490) mg/g in albuminuric KTRs (Supplemental Fig. S3*A*). In the CONTEXT study subgroups, KTRs with an increase in ACR were older and had a lower eGFR compared with controls (Table 1). Approximately 90% of KTRs in each of the three groups were treated with antihypertensives. Rejection before 3 mo was more prevalent (50%) in the ACR-increased group.

### Urine Complement Activation Split Products Are Elevated in Albuminuria

**Figure 1.**
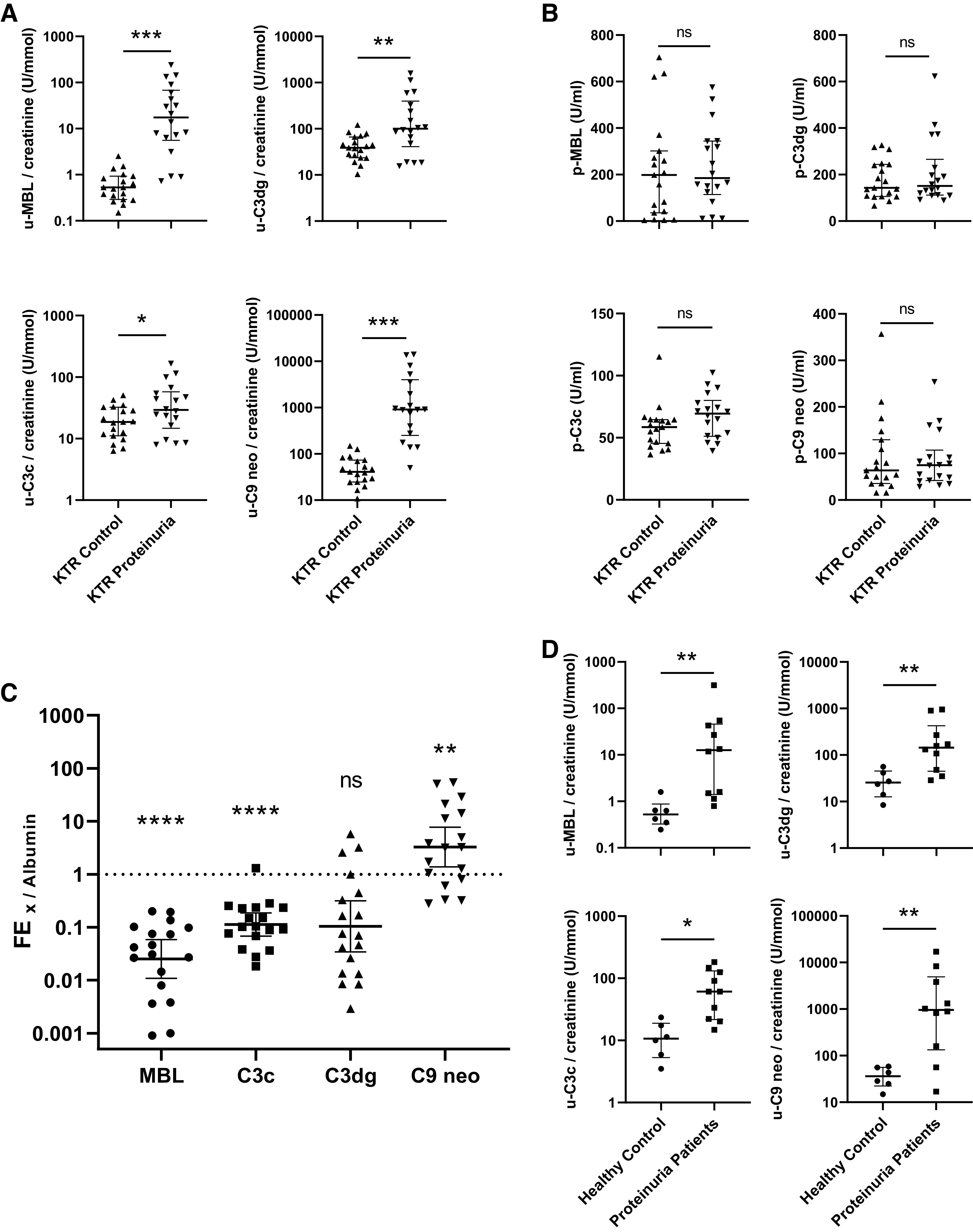
Urine excretion of activated complement is increased in albuminuria. *A* and *B*: creatinine-normalized spot urine (u; *A*) and plasma (p) levels (*B*) of mannose-binding lectin (MBL) and complement activation split products C3c, C3dg, and soluble (s)C5b-9-associated C9 neoantigen (C9 neo) in kidney transplant recipients (KTRs) with albumin-to-creatinine ratios (ACR) of ≥300 mg/g (*n* = 18) and <30 mg/g (*n* = 19). *C*: fractional excretion (FE) ratios of MBL, C3c, C3dg, and sC5b-9 to the FE of albumin. The dotted line marks the ratio of 1:1 to albumin with a significance from 1 by a one-sample Wilcoxon test. *D*: nontransplanted patients with proteinuria (*n* = 10) compared with nontransplanted healthy controls (*n* = 6). Data are shown as medians with interquartile ranges in *A*, *B*, and *D*. In *C*, bars represent geometric means with 95% confidence intervals. *P < 0.05, **P < 0.01, ***P < 0.001, and ****P < 0.0001. ns, not significant.

KTRs with ACRs of ≥300 mg/g had significantly higher urinary excretion of MBL, C3c, C3dg, and sC5b-9-associated C9 neoantigen (normalized to creatinine) compared with KTRs without albuminuria (Fig. 1*A*). In contrast, there were no differences between groups in the corresponding plasma samples (Fig. 1*B*). Plasma albumin was slightly but significantly lower in KTRs with albuminuria (Supplemental Fig. S3*B*). In KTRs with ACRs of ≥300 mg/g, MBL and C3c, but not C3dg, displayed lower FE than albumin (ratio < 1); the FE ratio of C9 neoantigen over albumin was 3.3 (geometric mean, 95% confidence interval: 1.4–7.8) and was significantly different from MBL (*P* < 0.001), C3c (*P* = 0.003), and C3dg (*P* < 0.001) by one-way ANOVA (Fig. 1*C*). Nontransplant patients from *cohort 3* with ACRs of ≥300 mg/g also had increased urine MBL-, C3c-, C3dg-, and C9 neoantigen-to-creatinine ratios compared with controls (Fig. 1*D*).

### C3dg and sC5b-9-Associated Neoantigen Excretion in KTRs Reflect Changes in Albuminuria Over Time

**Figure 2.**
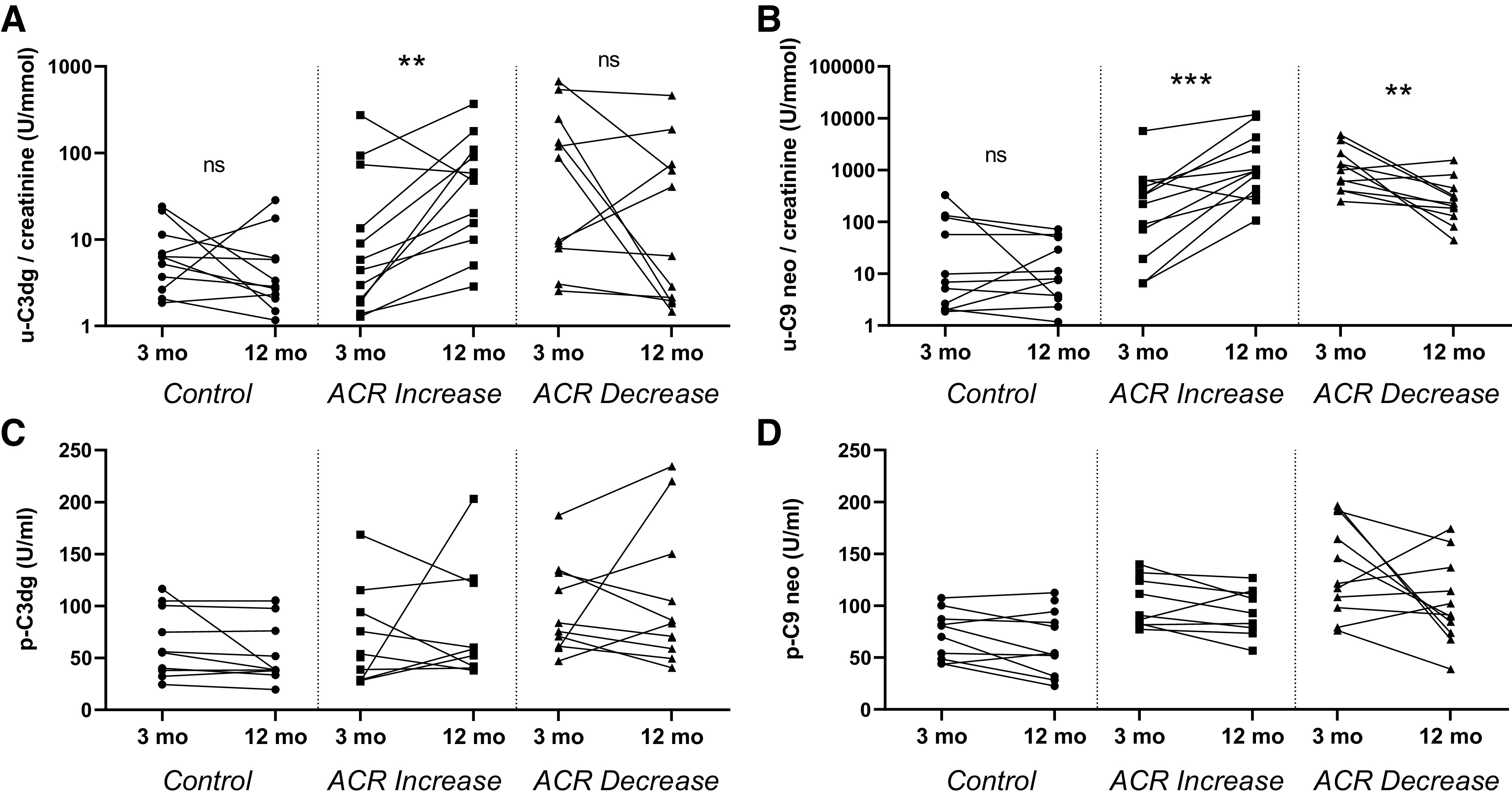
Complement excretion reflects changes in albuminuria. Creatinine-normalized urine (u) C3dg (*A*) and urine soluble C5b-9-associated C9 neoantigen (C9 neo; *B*) as well as plasma (p) concentrations of C3dg (*C*) and C9 neoantigen (*D*) in kidney transplant recipients (KTRs) from the CONTEXT study at 3 and 12 mo after transplantation. KTRs with stable albumin-to-creatinine ratio (ACR) of <30 mg/g (*n* = 11), an ACR increase of ≥30% (*n* = 12), or with an ACR decrease of ≥30% (*n* = 11) were selected from the CONTEXT study biobank database. **P < 0.01 and ***P < 0.001. ns, not significant.

Urine C3dg- and C9 neoantigen-to-creatinine ratios increased significantly from 3 to 12 mo in KTRs with a ≥30% increase in ACR, whereas levels did not change in KTR controls (Fig. 2*A*). KTRs with albuminuria at 3 mo and a ≥30% decrease in ACR at 12 mo had a significant decrease in C9 neoantigen (Fig. 2*B*) but not C3dg excretion (Fig. 2*A*). The absolute changes in ACR correlated significantly with the absolute changes in C3dg (*r* = 0.50, *P* = 0.003) and sC5b-9 (*r* = 0.79, *P* < 0.001; Supplemental Fig. S4). Plasma C3dg and C9 neoantigen concentrations did not change in any group (Fig. 2, *C* and *D*). eGFR was unchanged or increased in all groups regardless of urine albumin level (Table 1). Previous rejection was not significantly associated with urine complement excretion (Supplemental Fig. S5). The distribution of urine complement split products levels was unrelated to the original kidney disease in KTRs (Supplemental Fig. S6).

### Urine sC5b-9-Associated C9 Neoantigen but Not C3dg Concentration Correlate With Urine Protein Excretion

**Figure 3.**
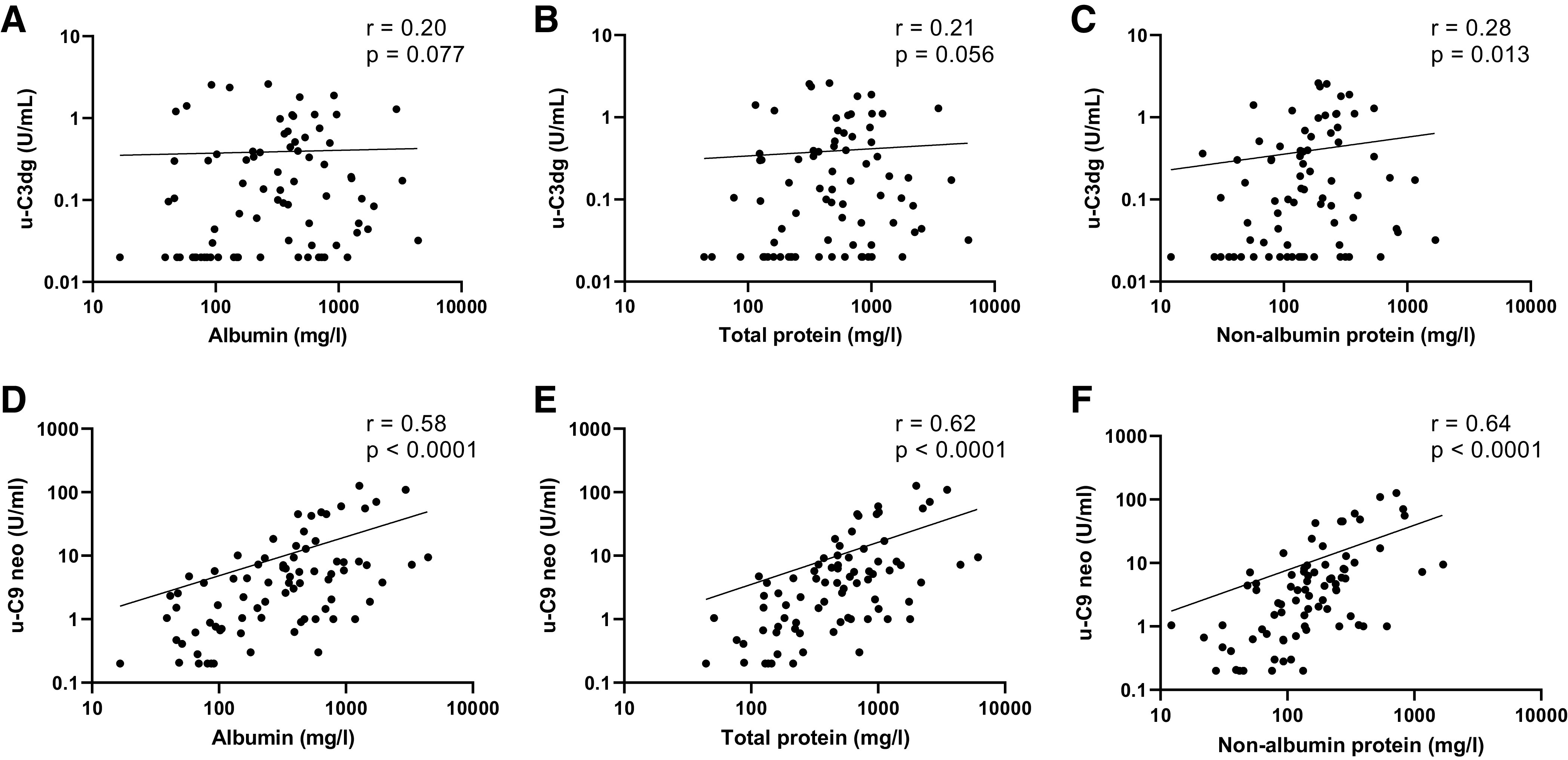
C9 neoantigen (C9 neo) correlates with protein in urine. Correlations between C3dg concentration and urine (u) albumin concentration (*A*) or urine total protein concentration (*B*) and nonalbumin protein concentration (*C*). Urine C9 neoantigen concentration correlated with albumin (*D*), total protein (*E*), and nonalbumin protein concentration (*F*). Spearman’s correlations were performed on pooled data from kidney transplant recipients (KTRs) with an albumin-to-creatinine ratio (ACR) of ≥300 mg/g from *cohort 1* (*n* = 18) patients with an ACR of >300 mg/g (*n* = 10); KTRs from CONTEXT study subgroups with an ACR increase and ACR decrease at both 3 mo (*n* = 23) and 12 mo (*n* = 23) are included. Three KTRs from the CONTEXT study that were analyzed but excluded because the change in ACR was <30% were also included (total *n* = 80). Log-log curves were fitted. Nonalbumin protein = total protein – albumin.

To indirectly assess if the urinary complement products represent aberrant filtration from plasma, we correlated urine albumin or protein and complement factor concentrations in urine samples from all patient groups (*n* = 80). C3dg did not correlate with urine albumin or total protein (Fig. 3, *A* and *B*) but only with nonalbumin protein (Fig. 3*C*). C9 neoantigen urine concentration correlated significantly with urine concentrations of albumin, total protein, and nonalbumin protein (Fig. 3, *D*–*F*).

### C9 Neoantigen Is Associated With uEVs Only in KTRs With Albuminuria

**Figure 4.**
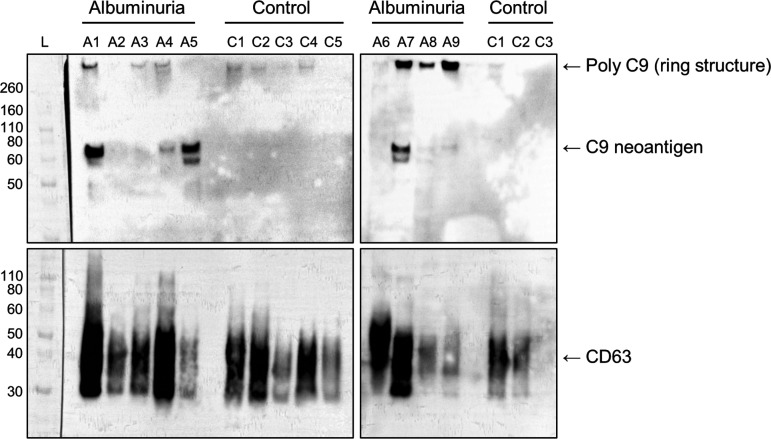
C5b-9-associated C9 neoantigen is present in urine extracellular vesicles (uEV) in posttransplant albuminuria. uEVs were isolated by ultracentrifugation at 220,000 *g* from kidney transplant recipients (KTRs) with an albumin-to-creatinine ratio of ≥300 mg/g (*n* = 9) and KTR controls (*n* = 8) from *cohort 1*. Samples were normalized to 0.4 mmol/L creatinine concentration by relative dilution in PBS and separated by gel electrophoresis. Samples were analyzed by Western blot analysis for C9 neoantigen (reducing conditions, expected molecular weight: 61 kDa) and the uEV marker CD63 (nonreducing conditions, expected molecular weight: 30–60 kDa due to glycosylated forms). A band corresponding to C9 neoantigen was present in albuminuric KTRs *A1*, *A5*, and *A7* and weakly in *A4*, *A8*, and *A9*. Polymeric C9 neoantigen bands were detected at >260 kDa in *A7*, *A8*, and *A9*, most likely representing ring structure C9 (i.e., C5b-9, SDS stabile when intact). Multiple bands are interpreted as degradation products carrying the C9 neoepitope. L, ladder.

The antibody directed against a C9 epitope exposed only after incorporation in a C5b-9 ring structure detected a protein migrating at the predicted size of 61 kDa (corresponding to monomer C9 neoantigen) and at >260 kDa (corresponding to intact C9 polymer ring) in serially diluted serum separated by SDS-PAGE only in the presence of primary antibody (Supplemental Fig. S7, *A* and *B*). A protein with a similar migratory pattern was detected in six of nine of creatinine-normalized uEV fractions from KTRs with albuminuria but was absent in KTRs without albumin in the urine (*n* = 8), whereas the uEV marker CD63 was present with the expected migratory pattern at 30–60 kDa in all nine KTRs with albuminuria and in seven of eight KTR controls (Fig. 4).

### C3 Activation Split Products and C5b-9-Associated C9 Neoantigen Are Present in uEVs From the Proximal Tubules

**Figure 5.**
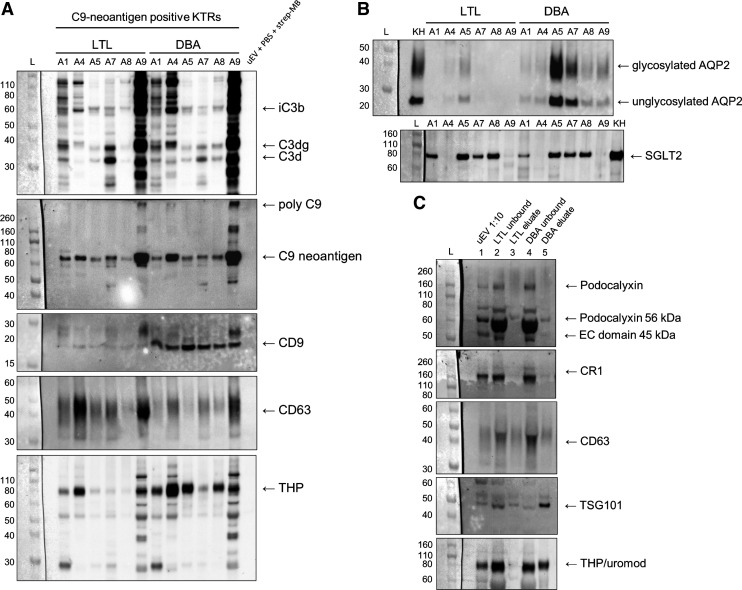
Complement activation split products are present in urine extracellular vesicles (uEVs) from the proximal tubules. Six C9 neoantigen-positive kidney transplant recipients (KTRs) were further investigated to localize a site of activation. Lectin affinity enrichment was performed on resuspended polyethylene glycol (PEG)-precipitated uEV pellets from KTRs *A1*, *A4*, *A5*, *A7*, *A8*, and *A9*. uEVs were enriched from proximal tubules by *Lotus tetragonolobus* lectin (LTL), which has affinity for fucose, and from collecting duct principal cells by *Dolichos biflorus* agglutinin (DBA), with affinity for *N*-acetylgalactosamine. uEV pool (from *A1*, *A4*, *A5*, *A7*, *A8*, and *A9*) incubated with PBS and streptavidin-conjugated magnetic beads (strep-MB) was included as a negative control. Enriched uEVs were normalized to volume (10 µL of sample in each well) and analyzed by Western blot analysis for iC3b (63 kDa)/C3dg (37 kDa)/C3d (33 kDa; polyclonal α-C3d), C9 neoantigen (61 kDa), CD9 (expected 25 kDa but migrating at ∼20 kDa), CD63 (30–60 kDa), and Tamm-Horsfall protein/uromodulin (THP/uromod; expected size 64 kDa; *A*); for AQP2 (appearing at 26 kDa and glycosylated at 35-45 kDa and for Na^+^-glucose transporter 2 (SGLT2; 77 kDa; *B*). *C*: representative blots from C9-positive KTRs showing that podocyte markers podocalyxin and complement receptor (CR1) were present in the unbound fractions but not in LTL and DBA eluates, whereas CD63 and tumor susceptibility gene 101 protein (TSG101; 44 kDa) were also present in the eluates. The sample was almost cleared from THP after LTL isolation, whereas DBA coisolated THP to a large extent. *Lane 1*: uEV pool from *A1*, *A4*, *A5*, *A7*, *A8*, and *A9* diluted 1:10; *lane 2*: unbound fraction after incubation with biotinylated LTL and streptavidin magnetic beads; *lane 3*: LTL eluate (fucose); *lane 4*: unbound fraction after DBA isolation; *lane 5*: DBA eluate (*N*-acetylgalactosamine). CD9, CD63, and CR1 samples were analyzed under nonreducing conditions. All other markers were run under reducing conditions. EC, extracellular; KH, 5 µg human kidney tissue homogenate.

C9 neoantigen-positive KTRs were further investigated to localize the cellular origin of complement deposition and to validate membrane association. Proximal tubule uEVs were isolated by LTL precipitation and collecting duct principal cell uEVs were isolated by DBA precipitation. Western blot analysis for C3d gave signals corresponding to the opsonins iC3b, C3dg, and C3d in several KTRs in both LTL- and DBA-isolated uEVs, and both LTL- and DBA-isolated uEVs were positive for C9 neoantigen in six of six samples (Fig. 5*A*). The uEV marker CD9 was present in all uEVs but was much more intense in DBA-isolated uEVs, whereas the uEV marker CD63 was more intense in LTL-isolated uEVs (Fig. 5*A*). The signal for THP was more intense in all DBA-isolated uEVs (Fig. 5*A*). AQP2 signals were much less intense in LTL-isolated uEVs than in DBA-isolated uEVs, whereas SGLT2 could be detected in both and with similar intensity in four of six samples (Fig. 5*B*). The podocyte markers podocalyxin and CR1/CD35 were present in the unbound fraction after both LTL and DBA isolates but were not enriched in their respective eluates (Fig. 5*C*). CD63 and TSG101 were detectable in the eluates and validate the presence of uEVs. LTL but not DBA eluate was relatively free of coisolated THP compared with the corresponding unbound fractions (Fig. 5*C*).

### C9 Coimmunoprecipitates C9 Neoantigen, SGLT2, and CD63

**Figure 6.**
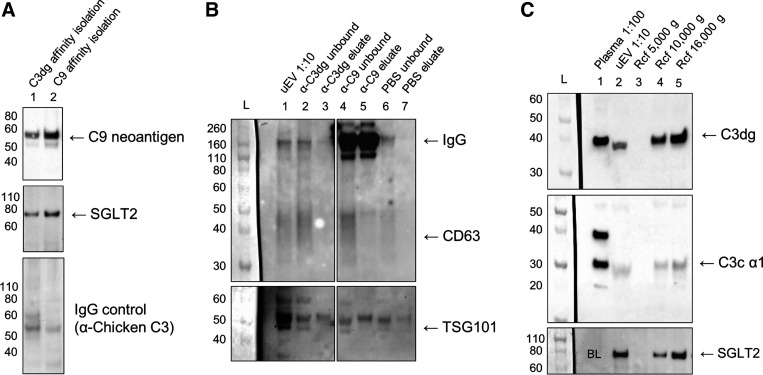
Immunoprecipitation for C3dg and C9 in urine extracellular vesicles (uEVs). Pooled polyethylene glycol (PEG)-precipitated uEVs from albuminuric kidney transplant recipients (KTRs) *A1*, *A4*, *A5*, *A7*, *A8*, and *A9* were subjected to secondary isolation by immunoaffinity for C3dg or native C9 or to secondary washing and centrifugation. *A*: uEV fractions enriched by immunoaffinity for C3dg (*lane 1*) and native C9 (*lane 2*) both coisolated C9 neoantigen and Na^+^-glucose transporter 2 (SGLT2). No corresponding bands were seen in the IgG control blot. *B*: Western blots showing that CD63 was coisolated with native C9 but not C3dg and that tumor susceptibility gene 101 protein (TSG101) was not coisolated by either antibody. *Lane 1*: uEV pool 1:10; *lane 2*: C3dg unbound fraction removed before washing of beads; *lane 3*: C3dg eluate; *lane 4*: C9 unbound fraction removed before washing; *lane 5*: C9 eluate; *lane 6*: PBS unbound fraction removed before washing; *lane 7*: PBS eluate. *C*: pooled uEVs were diluted in in PBS ∼1:12 and centrifuged for 60 min at a relative centrifugal force (rcf) of 16,000 *g*. The pellet was resuspended to the original volume, mixed, and divided in two fractions. Both fractions were diluted in PBS ∼1:50 and centrifuged for 30 min at either 10,000 *g* or 5,000 *g*, after which the pellets were again resuspended to twice its original volume. The crude urine was centrifuged at 5,000 *g* for 15 min before PEG precipitation, and samples are thus expected to be free of particles below this limit. Lanes are as follows: plasma 1:100 (*lane 1*), uEV pool 1:10 (*lane 2*), wash and centrifugation at 5,000 g (*lane 3*), wash and centrifugation at 10,000 *g* (*lane 4*), and wash and centrifugation at 16,000 g (*lane 5). Lane 1* is blank in the SGLT2 blot. CD63 samples were analyzed under nonreducing conditions; all other markers were reduced. L, ladder.

Affinity isolation performed on the C9-positive uEV pool using monoclonal α-C3dg or α-C9 (non-neoepitope) antibodies led to coimmunoprecipitation of C9 neoantigen and SGLT2 (Fig. 6*A*). CD63 and TSG101 were present in the unbound fractions after immunoprecipitation, whereas only C9 and not C3dg coprecipitated CD63 and none coprecipitated TSG101 (Fig. 6*B*).

### C3dg Is Associated With Large uEVs From the Proximal Tubules

The association of C3dg and SGLT2 with uEVs in the C9 neoantigen-positive uEV pool was further validated by secondary washing and centrifugation at 16,000 *g*, removing potential soluble protein contaminants that also precipitated by PEG (final concentration: 8%). Signals for C3dg and SGLT2 were present in the uEV pool diluted 1:10 and were maintained after the first wash and centrifugation at 16,000 *g*. Signals were also present after the second wash and centrifugation at 10,000 *g* but not at 5,000 *g* (negative control). The signal for C3dg in the uEV pool (1:10) was comparable to the signal for C3dg in plasma (1:100), whereas the signal for the C3c α1-chain was much weaker compared with the C3c α1-chain in plasma. The C3c α1-chain was also maintained after wash and centrifugation steps (Fig. 6*C*). The specificity of biotinylated rat IgM anti-huC3dg was validated by detection of a 37-kDa band in activated serum and plasma but not in C3-depleted serum. However, it could not be detected in the C9-positive uEV pool after LTL enrichment (Supplemental Fig. S7*C*).

**Figure 7.**
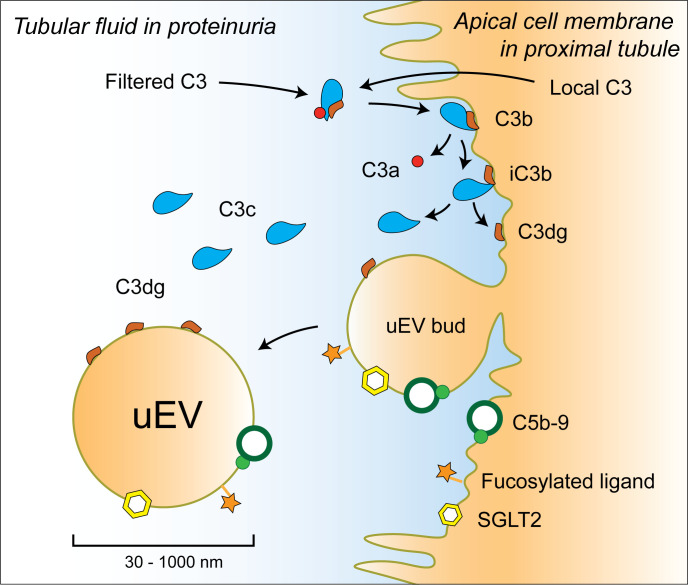
Illustration depicting the pattern of surface and fluid phase molecules observed in proximal tubular urine extracellular vesicles (uEV) and the mechanism for C3b/iC3b cleavage to C3c and C3dg on the proximal tubular apical membrane [inspired by Troldborg et al. (36)]. uEVs selected by affinity isolation for fucose and fucosylated ligands with *Lotus tetragonolobus* lectin (LTL) coisolated iC3b and C3dg, C9 neoantigen, and Na^+^-glucose transporter 2 (SGLT2) in albuminuric kidney transplant recipients. Coisolation by LTL of collecting duct and podocyte uEVs was minimal.

The pattern of molecules detected in LTL-precipitated, proximal tubular uEVs is shown in Fig. 7.

### Immunohistochemistry for C3d and C5b-9-Associated C9 Neoantigen Shows Apical Tubular Staining in Proteinuria

**Figure 8.**
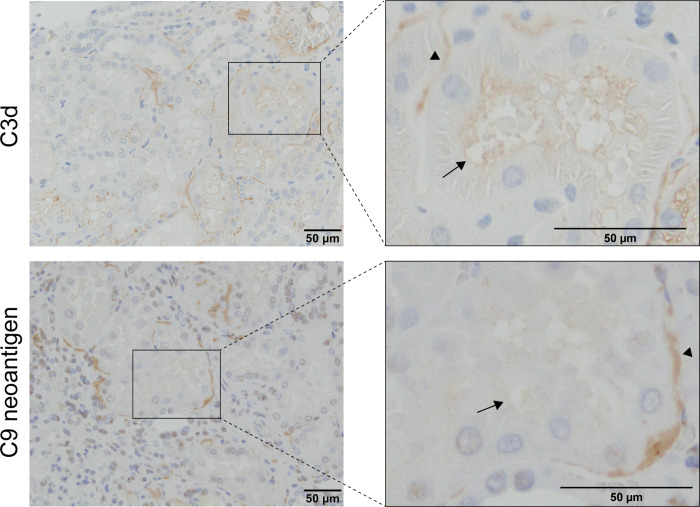
Tubular complement deposition in proteinuria. Tumor nephrectomy kidney tissue sections from patients with dipstick-verified proteinuria at the time of nephrectomy were stained for C3d (polyclonal rabbit anti-C3d, Cat. No. A0063, DAKO) and C9 neoantigen (monoclonal mouse anti-C9 neoantigen, WU13-15). In areas, tubular staining was detectable along apical (long arrow) and basolateral (short arrow) membranes. Original magnification: ×400.

Immunohistochemical labeling for C3d (polyclonal) and C9 neoantigen in nontumorous parts of cancer nephrectomy kidney tissue showed tubular staining in cortical tissue. Staining was observed in the luminal membranes of tubule cells as well as in tubular basement membranes (Fig. 8). For C3d, this pattern was observed both in nonproteinuric controls (Supplemental Fig. S8*A*) and in sections from patients with proteinuria at the time of nephrectomy (Supplemental Fig. S8*C*). C9 neoantigen was present mainly in the vascular endothelium and in the vascular pole of the glomeruli in nonproteinuric sections (Supplemental Fig. S8*D*). Some staining was observed in the tubular basement membrane also in controls, but this was much more pronounced in sections with proteinuria (Supplemental Fig. S8*C*), which also displayed staining of tubular cell luminal membranes (Fig. 8). Sections with proteinuria incubated with isotype control antibodies were negative for both C3d and C9 neoantigen (Supplemental Fig. S8, *B* and *E*).

## DISCUSSION

The present study shows that the urinary excretion of key complement activation split products was increased in albuminuria without concomitant changes in plasma levels and that changes in urinary excretion of C3dg and sC5b-9-associated C9 neoantigen were associated with changes in albuminuria over time. The elevated FE ratio of C9 neoantigen to albumin and MBL suggests postfiltration tubular generation. C9 neoantigen was enriched in proximal tubule-derived uEVs from KTRs with albuminuria and coprecipitated with C3 activation markers and SGLT2, with minimal coisolation of podocyte and collecting duct uEVs. This identifies membrane attack in the proximal tubules by nephron-specific uEV analysis.

In the normal glomerulus, protein sieving is largely a function of plasma concentration and molecular size, but it is also affected by conformation and charge (43). Given that sieving across a defect glomerular barrier is also size and structure dependent, the relative level of aberrantly filtrated molecules can be estimated. Plasma MBL with a molecular weight of 400–700 kDa would be expected to pass less than albumin (65 kDa), which was also observed. The C9 neoantigen occurs in plasma on C9 molecules incorporated in sC5b-9 (700–1,300 kDa), and intact sC5b-9 would also be predicted to filter much less than albumin. Interestingly, the calculated FE ratio for C9 neoantigen to albumin was greater than the FE ratio for the similarly sized MBL, and individual patients had a 10- to 50-fold higher ratio to albumin. This finding suggests a net addition of C9 neoantigen, most likely by activation and (s)C5b-9 ring formation from native C9, after filtration. The lower FE ratio for C3c (135 kDa) could be explained by less filtration or degradation by proteases (44, 45). Unfortunately, the addition of protease inhibitor cocktail was not added to these samples when the biobank was collected. Physiological filtration and tubular reabsorption of albumin are also confounding factors and are not taken into account by this approach (46, 47). Large net reabsorption of albumin would yield a falsely elevated FE ratio to albumin for any protein that is not reabsorbed along the tubules. However, this effect would be lower if albumin reabsorption was disrupted (e.g., in nephrotic syndrome) (47). Moreover, several complement precursors are like albumin ligands for megalin-cubilin receptors and thus also undergo endocytic uptake, and tubular cells and podocytes can synthetize C3, which might add to intratubular C3 (48, 49). Plasma albumin levels within the normal range indicate that liver synthesis function was intact.

To gain dynamic insights into apical complement deposition, we analyzed uEVs carrying membrane-associated proteins (Fig. 7). C9 neoantigen-positive uEVs were predominantly recovered from KTRs with albuminuria regardless of the method used for isolation, and lectins were used to enrich uEVs from specific nephron segments in C9-positive subjects. LTL and DBA have been shown to differentiate between SGLT2- and AQP2-carrying uEVs (18), and the C9 neoantigen signal was confirmed in all lectin-enriched uEVs. Bands corresponding to C3 activation split products were present in most samples but with different intensity and document the sequential activation of complement on tubular apical membranes. LTL-isolated uEVs were confirmed to be relatively free of contaminating THP and AQP2 (collecting duct marker) compared with DBA-isolated uEVs. The presence of THP could explain the presence of SGLT2 in DBA-isolated uEVs, as SGLT2 might be coexpressed with THP in some cells. Merging of vesicles could also explain this finding, and the fact that uEVs were obtained from transplanted kidneys with proteinuria and not normal healthy subjects should be kept in mind. We found a stronger signal for CD63 than CD9 in proximal tubular uEVs, whereas the opposite was observed in DBA-isolated uEVs. This indicates that the expression of these markers differs in different tubular segments, which is in accordance with findings by others (50, 51). LTL isolation seems highly specific and thus documents iC3b/C3dg and C9 neoantigen deposition in proximal tubular uEVs, whereas DBA isolates contain collecting duct uEVs but also more THP impurities and uEVs from other segments. Deposition in collecting duct uEVs can therefore not be verified.

CR1/CD35 binds C3b and with less affinity iC3b and promotes phagocytosis and clearance of opsonized particles (12). It is expressed on podocytes and is a marker of podocyte uEVs (52–54). Complement on uEVs might be activated already in the glomerulus and stems from podocytes. However, both LTL and DBA isolation showed minimal coisolation of CR1 and the podocyte marker podocalyxin, whereas CD63 and TSG101 signals were still present, strongly indicating that podocyte uEVs were not coisolated to a higher extent.

Complement activation occurs mainly on surfaces, and soluble forms in plasma and urine are surrogate measures that at best mirror surface events. Although complement could assemble directly on uEVs after release, existing immunohistochemical data have shown basolateral and apical tubular deposition of both C3dg and C5b-9 in human kidney biopsies (55, 56). Complement deposition also has predictive value for kidney allograft outcome (57). We observed apical tubular staining for C3d in both control tissue and subjects with proteinuria. The polyclonal antibody applied does not discriminate between native, nonactivated C3 and activation products C3b, iC3b, and C3dg that all carry C3d epitopes. Previously, the antibody gave negative tubular staining in acute tubular necrosis and significant positive staining in ischemia-reperfusion injury 48 h after transplantation in human subjects (58). Tubular staining for C5b-9 is often also found in controls and is associated to higher age and diseases such as cancer. Cancer nephrectomy control tissue might therefore not be appropriate as a control for complement activation products (56). More pronounced C5b-9 staining is often seen in proteinuria (56), however, which is in accordance with our immunohistochemical observations. Earlier studies have also reported apical staining in proteinuria but tended to regard this as an unspecific signal related to proteinuria (56). In rats with nephrotic range proteinuria, deposition of C5b-9 was predominantly apical, and inhibition of membrane attack complex formation was therapeutic (59).

C3c is released from iC3b to the fluid phase as C3dg remains on the cell membrane when C3 is deposited. Therefore, C3c should not be abundant in uEVs with C3dg deposition (but present in the fluid phase, as observed). Methods to differentiate between C3 split products in patients are lacking (60). Here, we addressed this problem using neoepitope-specific antibodies against C3c and C3dg, directed to sites only exposed after C3dg is released (14, 15, 36). Repeated wash and gradient centrifugation steps increased the likeliness that the C3dg signal was associated with larger vesicles of >100 nm (39) and not unspecific soluble protein conglomerates (38). However, the referred-to studies were performed in cell medium and results cannot be directly transferred to urine.

The source of complement activation is not addressed in this study, but luminal tubular complement activation could be augmented or directly mediated by other aberrantly filtered proteases, for example, thrombin (23, 61) or renin, recently discovered to activate C3 (62). Tubular dysfunction could lead to cellular “stress” and changed carbohydrate surface signatures detected by pattern recognition molecules, as documented in ischemia-reperfusion (63–65). This two-step pathway could also explain why isolated podocyte dysfunction in, for example, minimal change disease does not automatically lead to increased tubular C5b-9 deposition and poor kidney outcome. Stressed tubular cells in combination with proteinuria might be necessary for driving intratubular complement activation and progressive kidney injury over time.

In conclusion, the present data suggest that the apical surface of the tubular epithelium is exposed to and attacked by circulating complement components in direct relation to the degree of filtration barrier injury. Aberrantly filtered complement precursors are activated in tubular fluid and on tubular membranes and activation can be quantified in urine. The clinical significance for renal outcome is not addressed in this study and remains to be elucidated.

### Perspectives and Significance

We demonstrated that lectins can be used to select uEVs specifically from the proximal tubules and to document complement attack. Noninvasive uEV analysis could be useful as a diagnostic tool and for monitoring treatment regimens in the future. It can be speculated that intratubular complement activation may lead to maintenance of inflammation and ongoing complement attack, which could at least partially account for the ongoing epithelial injury and adverse kidney outcome associated with proteinuria. This concept is probably not restricted just to posttransplant proteinuria but may extend to proteinuria and CKD in general. Complement inhibitors reaching the intratubular space could potentially slow the progression of CKD in proteinuria.

## SUPPLEMENTAL DATA

10.6084/m9.figshare.15097095.v1 Supplemental Figs. S1–S8: https://doi.org/10.6084/m9.figshare.15097095.v1.

## GRANTS

This work was supported by grants from the University of Southern Denmark, Odense University Hospital’s PhD fund, the Danish Research Council, the Novo Nordisk Foundation, the Karen Elise Jensen Foundation, the Danish Kidney Patient Association, the Lundbeck Foundation, the Danish Nephrological Society, the Swedish Medical Association, and A. P. Møller and wife Chastine Mc-Kinney Møllers Fond til Almene Formaal, Grosserer L.F. Foghts Fond, Aarhus University Hospital, and Aarhus University.

## DISCLOSURES

No conflicts of interest, financial or otherwise, are declared by the authors.

## AUTHOR CONTRIBUTIONS

G.L.I., G.R.H, N.V.K., R.Z., P.S., K.M., C.B., B.J., The CONTEXT Study Group, H.B., Y.P., and B.L.J. conceived and designed research; G.L.I., N.V.K., R.Z., H.S., The CONTEXT Study Group, H.B., Y.P., and B.L.J. performed experiments; G.L.I., M.B.N., G.R.H., N.V.K., H.S., H.B., Y.P., and B.L.J. analyzed data; G.L.I., M.B.N., P.S., K.M., C.B., B.J., The CONTEXT Study Group, H.B., Y.P., and B.L.J. interpreted results of experiments; G.L.I. prepared figures; G.L.I., Y.P., and B.L.J. drafted manuscript; G.L.I., M.B.N., G.R.H., N.V.K., R.Z., H.S., P.S., K.M., C.B., B.J., The CONTEXT Study Group, H.B., Y.P., and B.L.J. edited and revised manuscript; G.L.I., M.B.N., G.R.H., N.V.K., R.Z., H.S., P.S., K.M., C.B., B.J., The CONTEXT Study Group, H.B., Y.P., and B.L.J. approved final version of manuscript.
